# Reviewer acknowledgement 2013

**DOI:** 10.1186/1472-6963-14-67

**Published:** 2014-04-22

**Authors:** Helena C Flemyng

**Affiliations:** 1 BioMed Central, Floor 6, 236 Gray's Inn Road, London WC1X 8HB, UK

## Abstract

**Contributing reviewers:**

The editors of BMC Health Services Research would like to thank all our reviewers who have contributed to the journal in Volume 13 (2013).

## 

Jos Aarts

Netherlands

Gilbert Abiiro

Ghana

Aaron Abuosi

Ghana

Timothy Abuya

Kenya

Astrid Adams

United Kingdom

Richard Adanu

Ghana

Rachael Addicott

United Kingdom

Marian Ådnanes

Norway

Kingsley Agho

Australia

Sutapa Agrawal

India

Ferdinando Agresta

Italy

Emmanuel Nwabueze Aguwa

Nigeria

Irene Akua Agyepong

Ghana

Maryam Ahmadian

Malaysia

Syed Masud Ahmed

Bangladesh

Riitta Ahonen

Finland

Collins K Ahorlu

Ghana

Bridget Aidam

United States of America

Tanimola Akande

Nigeria

Noori Akhtar-Danesh

Canada

Halida Akhter

Bangladesh

Stella Akinleye

Nigeria

Ralitsa Akins

United States of America

Khaled Al Surimi

Saudi Arabia

Vassilis Aletras

Greece

Philip Alexander

United Kingdom

David Alfandre

United States of America

Margaret Allman-Farinelli

Australia

Javier Alvarez-Galvez

Spain

Sherif Amer

Netherlands

Samad Amini-Bavil-Olyaee

United States of America

Eugenia Amporfu

Ghana

F Anagnostopoulos

Greece

Henning Boje Andersen

Denmark

Robert Anderson

United States of America

Ruth A Anderson

United States of America

Anders Anell

Sweden

Rose Anorlu

Nigeria

Fernando Antonanzas

Spain

Jane Appleton

United Kingdom

Anwer Aqil

United States of America

Jesús Aranaz-Andrés

Spain

Greg Arling

United States of America

David Armstrong

Canada

Greg Armstrong

Australia

Britt Arrelov

Sweden

Augustine Asante

Australia

Arlene Ash

United States of America

John Ele-Ojo Ataguba

South Africa

Carolyn Audet

United States of America

Asa Audulv

Sweden

Ilona Autti-Rämö

Finland

Anthony Avery

United Kingdom

Japheth Awiti

Kenya

Asa Axelsson

Sweden

Olalekan Ayo-Yusuf

South Africa

Muhammad Ayub

Canada

Saira Azhar

Pakistan

Syed Khurram Azmat

Pakistan

Akira Babazono

Japan

Max Oscar Bachmann

United Kingdom

Franz G Bader

Germany

Wendy Baird

United Kingdom

Petra Baji

Hungary

Solome Kiribakka Bakeera

Uganda

Rachel Baker

United Kingdom

Roland Bal

Netherlands

Ileana Baldi

Italy

Kaushik Banerjee

Switzerland

Maggi Banning

United Kingdom

Jim Banta

United States of America

Lawrence Barat

United States of America

Sarah Barber

South Africa

Rosaline Barbour

United Kingdom

Alessandro Barchielli

Italy

Margaret Barnes

Australia

David Barnett

United Kingdom

Mohsen Barouni

Iran

Régis Barros

Brazil

Gillian Bartlett

Canada

Pelham Barton

United Kingdom

Hasan Basarir

United Kingdom

Robert Basaza

Uganda

Emma Bassett

Canada

Andrew Bastawrous

Kenya

David Bates

United States of America

Adalgisa Battistelli

France

Christopher Baugh

United States of America

Sebastian Baumeister

Germany

Fariborz Bayat

Iran

Thomas Becker

Germany

Helene Bednarsh

United States of America

Katrien Beeckman

Belgium

Les Beitsch

United States of America

Mulugeta Belay

Ethiopia

Nathaniel Bell

Canada

Cecelia Bellcross

United States of America

Abdel Bellou

United States of America

Ben Bellows

Kenya

Susan Benbow

United Kingdom

Susan Mary Benbow

United Kingdom

David Benton

Switzerland

David Ben-Tovim

Australia

Justin Benzer

United States of America

Marge Berer

United Kingdom

Dan Berlowitz

United States of America

Oscar Bernal

Spain

Gonzalo Bernal-Hoyos

Australia

Nadine Berndt

Netherlands

Mercedes Bern-Klug

United States of America

Larry H. Bernstein

United States of America

Stan Bernstein

United States of America

Graham Bickler

United Kingdom

Silvia Bigatti

United States of America

Claudio Bilotta

Italy

Alexander Bischoff

Switzerland

Annette Bishop

United Kingdom

Rakesh Biswas

Malaysia

Seiji Bito

Japan

Anne Mette Bjerkan

Norway

Oyvind Bjertnaes

Norway

Alden Blair

Canada

Karl Blanchet

United Kingdom

James Blando

United States of America

Matthijs Blankers

Netherlands

Andrew Blann

United Kingdom

Said Bodur

Turkey

Megan Bohensky

Australia

Mary Bollinger

United States of America

Omotayo Bolu

United States of America

Malcolm Bond

Australia

Maryline Bonnet

Switzerland

Matthias Bopp

Switzerland

Xavier Bosch-Capblanch

Switzerland

Thomas John Bossert

United States of America

Deborah Bowen

United States of America

Maria Boyce

Ireland

Jacqueline Boyle

Australia

Kayvan Bozorgmehr

Germany

Aleida Braaksma

Netherlands

Marek Brabec

Czech Republic

Jeffrey Braithwaite

Australia

Anke Bramesfeld

Germany

Ros Bramwell

United Kingdom

K. Bremner

Canada

John FP Bridges

United States of America

William Brieger

United States of America

Lucy Brindle

United Kingdom

Kaylee Britain

Australia

Diana Brixner

United States of America

Joanna Broad

New Zealand

Jurgen Broeren

Sweden

Helen Bromley

United Kingdom

Michelle Bronze

South Africa

Leonie S Brose

United Kingdom

Melissa Brouwers

Canada

Carolyn Brown

United States of America

Diane Brown

United States of America

Paul Brown

United States of America

Christine Brown Wilson

United Kingdom

Charles Bruneau

France

Wendy Bryant

United Kingdom

Maurice Bucagu

Switzerland

Brian Buck

Canada

Daniel Budnitz

United States of America

Bussma Bugis

United States of America

Alexis Bujold

Canada

Viola Burau

Denmark

Bo Burstrom

Sweden

Olivier Busch

Netherlands

Leslie Butt

Canada

Warwick Butt

Australia

Ganbat Byambaa

Mongolia

Dominique Anne-Michele Cadilhac

Australia

Lucero Cahuana-Hurtado

Mexico

Stefano Calciolari

Switzerland

Amaia Calderón-Larrañaga

Spain

Audrey Calderwood

United States of America

Chantal Camden

Canada

Michael Campbell

United Kingdom

Marjo Campmans-Kuijpers

Netherlands

Maureen Canavan

United States of America

Math Candel

Netherlands

Guangwen Cao

China

Marylou Cardenas-Turanzas

United States of America

Axel Carlsson

Sweden

Paolo Carraro

Italy

Jose Carrillo

Mexico

Grant Carrow

United States of America

Patricia Carter

Australia

Rob Carter

Australia

Marisa Casale

South Africa

James Cawley

United States of America

Clare Chandler

United Kingdom

Anne M Chang

Australia

Hsien-Yen Chang

United States of America

Yu-Chia Chang

Taiwan

Ming-Che Chao

Taiwan

Joanna Charles

United Kingdom

Joanna Chataway

United Kingdom

Himanshu Chaturvedi

India

Nasim Chaudhry

United Kingdom

Kath Checkland

United Kingdom

Jane Chege

Zambia

Julie Chen

Hong Kong

Liang-Kung Chen

Taiwan

Wen Chen

China

Yingyao Chen

China

Yu-Chun Chen

Taiwan

Alice Cheng

Canada

Ivor Chestnutt

United Kingdom

Chunhuei Chi

United States of America

Iris Chi

United States of America

Victor Chimhutu

Norway

Michael Chin

United States of America

Weng Yee Chin

Hong Kong

Shang-Jyh Chiou

Taiwan

Jennifer Chipps

Australia

Catherine Chittleborough

United Kingdom

Christopher Chong

Canada

Shin-Yi Chou

United States of America

Christos Chouaid

France

Eric Christensen

United States of America

Hannah Christensen

United Kingdom

Terkel Christiansen

Denmark

Ogoamaka Ifeagachukwu Chukwuogo

Nigeria

Kathryn Church

United Kingdom

Robyn Clark

Australia

Terryann Clark

New Zealand

Robyn Clay-Williams

Australia

Julie Cliff

Mozambique

Jacqueline Close

Australia

Virginie Cobigo

United Kingdom

Claudia Coeli

Brazil

Amy Cohen

United States of America

Raphael Cohen-Almagor

United Kingdom

Dirk Colaert

Belgium

Claire Collins

Ireland

G Colville

United Kingdom

Elizabeth Comino

Australia

John Connell

Australia

Luke Connelly

Australia

Coy Consuelo

Australia

Benjamin Cook

United States of America

Christina Cordero

United States of America

Tony Cornford

United Kingdom

Indalecio Corugedo

Spain

Jeralynn Cossman

United States of America

Massimo Costantini

Italy

Elizabeth Costenbader

United States of America

Francesc Cots

Spain

Angela Coulter

United Kingdom

Ian Couper

South Africa

Jason Coupet

United States of America

Elizabeth Cox

United States of America

Julie Cragg

United States of America

Peter Cram

United States of America

Jane Murray Cramm

Netherlands

Iain Crombie

United Kingdom

Paula Cronin

Australia

Luciane Cruz Lopes

Brazil

Greta Cummings

Canada

Frances Cunningham

Australia

Kay Currie

United Kingdom

Lesley Curtis

United States of America

Sufia Dadabhai

United States of America

Dave Dagnan

United States of America

Philip Ayizem Dalinjong

Ghana

Olivia Dalleur

Belgium

Yoswa Dambisya

South Africa

Olga Catherina Damman

Netherlands

Teresa Damush

United States of America

Denise D'Angelo

United States of America

Karen Daniels

South Africa

Andriy Danyliv

Ukraine

Granlund David

Sweden

Keith Davis

United States of America

Tere Dawson

Australia

Johanna De Almeida Mello

Belgium

Geertruida H. De Bock

Netherlands

Antoinette De Bont

Netherlands

Vincent De Brouwere

Belgium

Margaretha Christine De Bruijne

Netherlands

Jan De Lepeleire

Belgium

Koen De Meester

Belgium

Carl De Wet

United Kingdom

Katinka De Wet

South Africa

Reza Dehnavieh

Iran

Ellen Deilkås

Norway

Kim Delbaere

Australia

Derek Delia

United States of America

Nadia Demarteau

Belgium

Spiros Denaxas

United Kingdom

Sarah Dennis

Australia

Linley Denson

Australia

Mike Dent

United Kingdom

Kebede Deribe

Ethiopia

John Colin Harris Dewdney

Australia

Anne Dezetter

Canada

Kumar Dharmarajan

United States of America

Mirko Di Rosa

Italy

Papa Alassane Diaw

Senegal

Virginia Dickson-Swift

Australia

Peter Dieckmann

Denmark

Marjolein Dieleman

Netherlands

Joakim Dillner

Sweden

Nafees Din

United Kingdom

Yan Ding

Germany

Kylie Dingwall

Australia

Francois Dionne

Canada

Simon Dixon

United Kingdom

Priscilla Dlamini

Swaziland

Mai Do

United States of America

Hengjin Dong

China

Amol Dongre

India

Claire Donnellan

Ireland

Diane Doran

Canada

Tim Doran

United Kingdom

Nadine Dougall

United Kingdom

Michelle Dowsey

Australia

Alizon Draper

United Kingdom

Alex Dregan

United Kingdom

Arik Drucker

Canada

Frances Drummond

Ireland

María Beatriz Duarte Gómez

Mexico

Dominique Dubourg

Belgium

Stephen Duckett

Canada

Martin Duerden

United Kingdom

Sonia Duffy

United States of America

James Dunbar

Australia

David Dunger

United Kingdom

Rachael Dunlop

Australia

Kenrik Duru

United States of America

Gilles Dussault

Portugal

Susan Dutton

United Kingdom

Anna-Karin Elisabeth Dykes

Sweden

Anne-Kirstine Dyrvig

Denmark

Marc Eckstein

United States of America

Karen Edmond

Australia

Jane Edwards

Australia

Nigel Edwards

United Kingdom

Arne H Eide

Norway

Kiran Ejaz

Pakistan

Elizabeth Ekirapa-Kiracho

Uganda

Eva Ekvall Hansson

Sweden

Ann Catrine Eldh

Sweden

Fadi El-Jardali

Lebanon

Carol Hall Ellenbecker

United States of America

Jacobi Elliott

Canada

Mark Emberton

United Kingdom

Brian Emerson

Canada

Jane Chinelo Enemuoh

Nigeria

Doortje Engel

Switzerland

Mike English

Kenya

Ethem Erginoz

Turkey

Ahmed Esmael

Ethiopia

Marie-Louise Essink-Bot

Netherlands

Beverley Essue

Australia

Jason Etchegaray

United States of America

Arash Etemadi

Iran

Enyi Etiaba

Nigeria

Catrin Evans

United Kingdom

Elizabeth Evans

United Kingdom

Joshua Evans

Canada

Boniface Ikenna Eze

Nigeria

Soludo Eze

Nigeria

Nkoli Ezumah

Nigeria

Marjan Faber

Netherlands

Maria Pia Fantini

Italy

Saeed Farooq

Pakistan

Maryam Farooqui

Malaysia

Patricia Fast

United States of America

Thomas Faunce

Australia

Arild Faxvaag

Norway

Ugo Fedeli

Italy

Frank Feeley

United States of America

Rayah Feldman

United Kingdom

Angeline Ferdinand

Australia

Laura Ferguson

United Kingdom

Montserrat Ferrer

Spain

Angelika Fex

Sweden

Guido Filler

Canada

Ulla Harriet Finne-Soveri

Finland

Kevin Fiscella

United States of America

Michael Fischer

United States of America

Laura Fish

United States of America

Margaret Fitch

Canada

Steffen Flessa

Germany

Margot Fleuren

Netherlands

Osnat Fliess Douer

Israel

Jan Florin

Sweden

Lindsay Forbes

United Kingdom

Jay Ford

United States of America

Roberto Forero

Australia

Yvonne Forsell

Sweden

Marianna Fotaki

United Kingdom

Anneke Leijntje Francke

Netherlands

Richard Frankel

United States of America

Bryony Dean Franklin

United Kingdom

Penelope Franklin

United Kingdom

Ellen Freiberger

Germany

Atle Fretheim

Norway

Eva Frey

Austria

Jan C. Frich

Norway

Christine Friestad

Norway

Bianca Frogner

United States of America

Jeffrey Fuller

Australia

Martha Funnell

United States of America

Sander Gaal

Netherlands

Marie-Pierre Gagnon

Canada

Jenny Gallagher

United Kingdom

Moses Galukande

Uganda

John-Michael Gamble

Canada

Javier Garcia-Campayo

Spain

Zoe Garrett

United Kingdom

Robin Gauld

New Zealand

Anna Gavin

United Kingdom

Angèle Gayet-Ageron

Switzerland

Diederike Geelhoed

Mozambique

Paul Wenzel Geissler

Norway

Paul Gemmel

Belgium

Andrew Georgiou

Australia

Max Geraedts

Germany

Oke Gerke

Denmark

Georgina Gethin

Ireland

Ana Gil Lacruz

Spain

Mark Gilchrist

United Kingdom

Luis Andrés Gimeno-Feliu

Spain

Michael Gionfriddo

United States of America

Dorota Girard

France

Alan Girling

United Kingdom

Richard Glazier

Canada

Deborah K Glencross

South Africa

Kate Goddard

United Kingdom

Brian Godman

Sweden

Katja Goetz

Germany

Lisa Gold

Australia

Rachel Gold

United States of America

Joanne Goldman

Canada

David Goldsbury

Australia

Adam Goldstien

United States of America

Barbara Gomes

United Kingdom

Patama Gomutbutra

Thailand

Daniela Goncalves

United Kingdom

Eduardo Gonzalez-Pier

Mexico

Phil Gooch

United Kingdom

David Goodrich

United States of America

Dawn Goodwin

United Kingdom

Jennifer Gosling

United Kingdom

Markus Grabka

Germany

Jonathan Graffy

United Kingdom

Chris Graham

United Kingdom

Julian Grant

Australia

Alex Graveling

United Kingdom

Darryl Gray

United States of America

Lee Green

Canada

Sheila Greenfield

United Kingdom

Joanne Greenhalgh

United Kingdom

Trisha Greenhalgh

United Kingdom

Michelle Greiver

Canada

Stefan Greß

Germany

Heather Gridley

Australia

Mark Griffin

Australia

Michel Grignon

Canada

Peter Grimison

Australia

Antonello Grippo

Italy

Oliver Groene

United Kingdom

Peter Groenewegen

Netherlands

Erik Groessl

United States of America

Vigdis Abrahamsen Grøndahl

Norway

Scott Grosse

United States of America

Francesco Grossi

Italy

Francisco Gude

Spain

Andrea Guerrini

Italy

P. Cristian Gugiu

United States of America

Janice Gullick

Australia

Martin Gulliford

United Kingdom

Freedom Nkhululeko Gumedze

South Africa

Jeff Guo

United States of America

Hari Om Gupta

India

Ipek Gurol-Urganci

United Kingdom

Brian Gushulak

Canada

Reinhard Guss

United Kingdom

Rikke Gut

Denmark

Iñaki Gutierrez

Spain

Stella Gwini

Australia

Marwan Habiba

United Kingdom

Karen Hacker

United States of America

Emina Hadziabdic

Sweden

Susanne Haga

United States of America

Hildi Hagedorn

United States of America

Terje P Hagen

Norway

Amy Hagopian

United States of America

Katja Marja Hakkarainen

Sweden

Claire Hale

United Kingdom

Carmen Hall

United States of America

Robert Hall

Australia

Dagmar Haller

Switzerland

Emma Halliday

United Kingdom

Antje Hammer

Germany

Bradley Hammill

United States of America

Euna Han

South Korea

Amresh Hanchate

United States of America

Nguyen Hang

Viet Nam

Philip Hannaford

United Kingdom

Jan-Cedric Hansen

France

Johan Hansen

Netherlands

Rachel Hardeman

United States of America

Jane Harries

South Africa

Jennifer Harris

United States of America

Mark Harris

Australia

Paul Harris

United States of America

Michael Harrison

United States of America

Christine Hartmann

United States of America

M Harun-Or-Rashid

Japan

Rowan Harwood

United Kingdom

Tomonori Hasegawa

Japan

Karen Hassell

United Kingdom

Felicity Hasson

United Kingdom

Henna Hasson

Sweden

Juanita Hatcher

Canada

Leslie Hausmann

United States of America

Helen Hawley-Hague

United Kingdom

Alison Hayes

Australia

Jamie Hayes

United Kingdom

Jean Hay-Smith

New Zealand

Mark Hayward

United Kingdom

Craig Anne Heflinger

United States of America

Frode Heldal

Norway

Maria Hellbom

Sweden

Emily Jacqueline Henderson

United Kingdom

Julie Henderson

Australia

Michelle Hendriks

Netherlands

Jane Hendy

United Kingdom

Bernadette Hensen

United Kingdom

Melissa Hensley

United States of America

Tarja Heponiemi

Finland

Maria Teresa Herdeiro

Portugal

Michael Herdman

Spain

R. Hermens

Netherlands

Katrin Hertrampf

Germany

Andrew Herxheimer

United Kingdom

Lisa Hess

United States of America

Sarah Hewlett

United Kingdom

Peter Heywood

Australia

Judith Hibbard

United States of America

Agnes Higgins

Ireland

Ann Hill

United Kingdom

Peter Hill

Australia

Ida Hilmi

Malaysia

Wolfgang Himmel

Germany

Hubertus Himmerich

Germany

Sven Gudmund Hinderaker

Norway

Kate Hinds

United Kingdom

Bradford Hirsch

United States of America

Peter Hjertholm

Denmark

Helena Hnilicová

Czech Republic

Michael Ho

United States of America

Wen-Hsien Ho

Taiwan

Barbara Hoffmann

Germany

Dag Hofoss

Norway

Margaret Hogan

Tanzania

Anne Hogden

Australia

Christa Hojlo

United States of America

Theresa Hoke

United States of America

Diane Holland

United States of America

Monika Hollander

Netherlands

Soren Holm

United Kingdom

Olaf Holmboe

Norway

Margaret Holmes-Rovner

United States of America

Ian Holt

United Kingdom

Caroline Homer

Australia

Matthew Hopcraft

Australia

Michael Horberg

United States of America

Frances Horgan

Ireland

Jennifer Horney

United States of America

David Howard

United States of America

Ya-Seng Hsueh

Australia

Hsiao-Yun Hu

Taiwan

Shanlian Hu

China

Hsiu-Chen Huang

Taiwan

Wei Huang

United States of America

Ruth Hubbard

Australia

Patrick Hudson

Netherlands

Peter Hudson

Australia

Whady Hueb

Brazil

Marco Huesch

United States of America

Jacqueline Hugtenburg

Netherlands

Robbert Huijsman

Netherlands

Nicholas Hulbert-Williams

United Kingdom

John Humphreys

Australia

Matthias Hunger

Germany

Dereck Hunt

Canada

Samia Hurst

Switzerland

Nusrat Husain

United Kingdom

Andrew Hutchings

United Kingdom

Allen Hutchinson

United Kingdom

Marie Hutchinson

Australia

Michael Hutchinson

Ireland

Raymond Hutubessy

Switzerland

Jee-In Hwang

South Korea

Emily Hyle

United States of America

Ilene Hyman

Canada

Rick Iedema

Australia

Kashef Ijaz

United States of America

Efthymios Iliopoulos

Greece

Namagembe Imelda

Uganda

Kazuhiko Inoue

Japan

Michelle Irving

Australia

Inyang Isong

United States of America

Hiroto Ito

Japan

Hilde Hestad Iversen

Norway

Angela Ives

Australia

Susan Jack

Canada

Claire Jackson

Australia

Sally Jacobs

United Kingdom

Gro Jamtvedt

Norway

Suze Jans

Netherlands

Jesse Jansen

Australia

Maria Jansen

Netherlands

Christian Janssen

Germany

Ingrid Janssen

Netherlands

Barbara Janssen-Solberg

Netherlands

Edward Janus

Australia

Anu Järvensivu

Finland

PN Roopalekha Jathanna

India

Ravi Jayadevappa

United States of America

Pinel Jean-François

France

Lianne Jeffs

Canada

Anupam Jena

United States of America

Rachel Jenkins

United Kingdom

Larissa Jennings

United States of America

Jakob Jensen

United States of America

Yun-Hee Jeon

Australia

Elisabeth Jeppesen

Norway

David Jimenez

Spain

Ingrid H Johansen

Norway

Emily Jones

United States of America

Fiona Jones

United Kingdom

Stephanie Jones

United Kingdom

Jimmy Jose

Oman

Nicole Juffermans

Netherlands

Kyoungrae Jung

United States of America

Kunal Kadakia

United States of America

Kumud Kafle

Nepal

Natan Kahan

Israel

John Kalbfleisch

United States of America

Karin Källander

Uganda

Lalit Kalra

United Kingdom

Dorcas Kamuya

Kenya

Eila Kankaanpää

Finland

Warren Kaplan

Switzerland

Charles Amnon Sunday Karamagi

Uganda

Staffan Karlsson

Sweden

Jon Karnon

Australia

Hitomi Kataoka

Japan

Gohei Kato

Japan

Noriko Kato

Japan

Hugo Katus

Germany

Abby Kazley

United States of America

Khawar Kazmi

Pakistan

Robin Kearns

New Zealand

Margaret Kelaher

Australia

Helen Keleher

Australia

Andrea Kent

Canada

Stephen Kerr

Australia

Paula Kersten

United Kingdom

Saif Khairat

United States of America

M. Mahmud Khan

United States of America

Nandita Khera

United States of America

Ai Leng Khoo

Singapore

Juliet Kiguli

Uganda

Cherry Kilbride

United Kingdom

Meelee Kim

United States of America

Sun-Young Kim

United States of America

Yoon Kim

South Korea

John Kinsman

Sweden

Patricia Kipnis

United States of America

Joses Muthuri Kirigia

Congo

Lars Kjellin

Sweden

Egil Kjerstad

Norway

Scott Klarenbach

Canada

Ruth M. Kleinpell

United States of America

Marianne Klemp

Norway

Catherine Klersy

Italy

Andrew Knighton

United States of America

Birthe Loa Knizek

Norway

Simo Kokko

Finland

Michaela Kolbe

Switzerland

Ryuichi Komatsu

Switzerland

Thomas R. Konrad

United States of America

Sascha Köpke

Germany

Ross Koppel

United States of America

Catherine Korachais

Belgium

Rosemary Korda

Australia

Sheri Koshman

Canada

Meera Kotagal

United States of America

Dhwani Kothari

United States of America

Marius Brostrøm Kousgaard

Denmark

Karl Krajic

Austria

Graham Kramer

United Kingdom

Allan Krasnik

Denmark

Søren Rud Kristensen

United Kingdom

Jimmie Kristensson

Sweden

Marielle Kroese

Netherlands

Edeltraut Kröger

Netherlands

Steinar Krokstad

Norway

Madelon Kroneman

Netherlands

Janet Krska

United Kingdom

Annemarije Kruis

Netherlands

Margaret Elizabeth Kruk

United States of America

Elizabeth Krupinski

United States of America

Marie Kruse

Denmark

Leighton Ku

United States of America

Wolfgang Kuenzel

Germany

Tendesayi Kufa

South Africa

Hakan Kulacoglu

Turkey

Sanjeewa Kularatna

Australia

Jeffrey Kullgren

United States of America

Kerry Kuluski

Canada

Chandan Kumar

India

Ramesh Kumar

Pakistan

Ludwig Kuntz

Germany

Dennis Kuo

United States of America

Yong-Fang Kuo

United States of America

Joseph Kutzin

Switzerland

Kari Kvaerner

Norway

Tracey-Lea Laba

Australia

Ronald Labonte

Canada

Claas Lahmann

Germany

Miriam Lainer

Austria

Christiaan Lako

Netherlands

Michel Landry

United States of America

Annette Lane

Canada

Peter Langhorne

United Kingdom

Paul Langley

United States of America

Holly Lanham

United States of America

Steven Lannoo

Belgium

Samuel Lapkin

Australia

Allison Larg

Australia

Andrew Larner

United Kingdom

Ann Larson

Australia

Karen Lasser

United States of America

Susan Latter

United Kingdom

Francis Lau

Canada

Jorgen Lauridsen

Denmark

Carl Lavie

United States of America

David Lawrence

Australia

Rod Lawson

United Kingdom

Simone Lazzini

Italy

Frederic Le Marcis

France

Jae-Ho Lee

South Korea

Sook-Jeong Lee

South Korea

Yu-Chen Lee

United Kingdom

France Légaré

Canada

Mengistu Legesse

Ethiopia

Sandra Leggat

Australia

David Legge

Australia

Juhani Lehto

Finland

Margus Lember

Estonia

Diana Lennon

New Zealand

Ryan Lennon

United States of America

Mondher Letaief

Tunisia

William Levack

New Zealand

Carol Levin

United States of America

Guohong Li

China

Ian Li

Australia

Shixue Li

China

Ya-Hsin Li

Taiwan

Ying-Chun Li

Taiwan

Wee Liang En Ian

Singapore

Maureen Lichtveld

United States of America

Sharon Licqurish

Australia

Jerker Liljestrand

Cambodia

Chung-Wei Christine Lin

Australia

Yu-Hua Lin

Taiwan

Anne Karin Lindahl

Norway

Suzanne Linder

United States of America

Stefan Lindgren

Sweden

Richard Lindley

Australia

Wendy Lipworth

Australia

Pascale Lissouba

France

Peter Littlejohns

United Kingdom

George Liu

Australia

Kawika Liu

United States of America

Su Liu

Hong Kong

Xiaoyun Liu

China

Xuefeng Liu Liu

United States of America

Yawen Liu

United States of America

George Lodge

United Kingdom

Benjamin Loevinsohn

United States of America

Yoon Kong Loke

United Kingdom

Carl Lombard

South Africa

Theodore Long

United States of America

Joanne Lord

United Kingdom

Karl Lorenz

United States of America

Valerie R. Louis

Germany

Jordan Louviere

Australia

Lee-Fay Low

Australia

Julia Lowe

Canada

Anne Lowell

Australia

Hong Lu

China

Lisa Lubomski

United States of America

Ramon Luengo-Fernandez

United Kingdom

Pisake Lumbiganon

Thailand

Juma Lungo

Tanzania

Kalevi Luoma

Finland

Courtney Lyles

United States of America

Georgios Lyratzopoulos

United Kingdom

Marion Maar

Canada

Stefanie Mache

Germany

Shingai Machingaidze

South Africa

James Macinko

United States of America

Kate Macintyre

Kenya

Lynette Mackenzie

Australia

Timothy Mackey

United States of America

Jean Macq

Belgium

Jose Maessen

Netherlands

Parker Magin

Australia

Nicola Magnavita

Italy

Pranitha Maharaj

South Africa

Christine Maheu

Canada

Elham Mahmoudi

United States of America

Tomas Mainil

Belgium

Gregory Makris

United Kingdom

Robert Malkin

United States of America

Stephen Maluka

Tanzania

Sandra Mandic

New Zealand

Jill Manthorpe

United Kingdom

Javier Mar

Spain

Geoff March

Australia

Bruno Marchal

Belgium

Paul Marschall

Germany

Marta Marsilio

Italy

Graham Martin

United Kingdom

Iñaki Martin Lesende

Spain

Jose Martin-Moreno

Spain

Steven Marwaha

United Kingdom

Helen Mason

United Kingdom

Thomas Mason

United Kingdom

Imrana Masroor

Pakistan

Tsitsi Masvawure

Uruguay

Mario Dinis Mateus

Brazil

Lauren Matheson

United Kingdom

Klazien Matter-Walstra

Switzerland

Dileep Mavalankar

India

Panagiotis Mavros

United States of America

Esther May

Australia

Serge Mayaka

Belgium

Eckhard Mayer

Germany

Philip Mayles

United Kingdom

Nicholas Barron Mays

United Kingdom

John Mazuski

United States of America

Anthony Mbonye

Uganda

Sheena McHugh

Ireland

Kevin McNamara

Australia

Nikki McCaffrey

Australia

Sara McEwen

Canada

Meghan McGrady

United States of America

Elizabeth McInnes

Australia

Diane McIntyre

South Africa

Meredith McIntyre

Australia

Sarah McIntyre

Australia

Michael McKee

United States of America

James McKinney

United States of America

Brian McKinstry

United Kingdom

John McKnight

United Kingdom

Barbara McPake

United Kingdom

Kathryn McPherson

New Zealand

Ian Stewart McRae

Australia

Graham Meadows

Australia

Ateev Mehrotra

United States of America

Devidas Menon

Canada

Joseph Mensah

Canada

Emmanouil Mentzakis

United Kingdom

Nicolas Menzies

United States of America

Mathew Mercuri

Canada

Lisa Merry

Canada

Sonja Merten

Switzerland

Jennifer Mertens

United States of America

Nadine Meskens

Belgium

Amy Metcalfe

Canada

Hongyu Miao

United States of America

James Middleton

Australia

Sandy Middleton

Australia

Patrik Midlöv

Sweden

Baukje Miedema

Canada

Cathy Mihalopoulos

Australia

Blanca-Tamayo Milagrosa

Spain

Marijana Miler

Croatia

Jeremy Miles

United Kingdom

Anton Miller

Canada

Christopher Miller

United States of America

Mirella Minkman

Netherlands

Karin Minnie

South Africa

Jose Joaquin Mira

Spain

Marie Misso

Australia

Patriek Mistiaen

Netherlands

Leander Mitchell

Australia

Oskar Mittag

Germany

Jessica Mittler

United States of America

Motlatso Gladys Mlambo

South Africa

Ebrahim Mohammed

Ethiopia

Khaled Mohammed

United States of America

Shafiu Mohammed

Germany

David Mohr

United States of America

Mohammed Mohsin

Australia

Sandra Moll

Canada

Andrew Molodynski

United Kingdom

Maureen Monaghan

United States of America

Lindsey Monteith

United States of America

Marjory Moodie

Australia

Rachael Moorin

Australia

Andrew Morden

United Kingdom

Michel Moreau

Belgium

Mike Morgan

Australia

Myfanwy Morgan

United Kingdom

Amani Thomas Mori

Tanzania

Christine Morris

Australia

Jacqui Morris

United Kingdom

Davide Mosca

Kenya

Grete Moth

Denmark

Jin Mou

Canada

Sandra Mounier-Jack

United Kingdom

Vladimir Mouraviev

United States of America

Cheryl Moyer

United States of America

Gemini Mtei

Tanzania

Godfrey Mubyazi

Tanzania

Stephanie Mueller

United States of America

Patrobas Mufubenga

Uganda

Ruben Mujica-Mota

United Kingdom

Judy Mullan

Australia

Artur Manuel Muloliwa

Mozambique

Michael Aloyce Munga

Tanzania

Zachary Munn

Australia

Carolyn Murray

Australia

Portia Mutevedzi

South Africa

David Muthaka

Kenya

Monde Muyoyeta

Zambia

Germano Mwabu

Kenya

Nancy Mwange

Kenya

Juliet Nabyonga Orem

Uganda

Lucio Naccarella

Australia

Farooq Naeem

Pakistan

Somil Nagpal

India

Nisha Naicker

South Africa

Anthony Naimi

Canada

Kavita Nair

United States of America

Gorrette Nalwadda

Uganda

Susan Nancarrow

Australia

Mabel Nangami

Kenya

Amol Navathe

United States of America

Richard Neal

United Kingdom

Fulvio Nedel

Brazil

Adrianne Nelson

United States of America

Maria Ines B. Nemes

Brazil

Duncan Neuhauser

United States of America

Peter Newcombe

Australia

David Newlands

United Kingdom

Zekariyas Nezenega

Ethiopia

Surachat Ngorsuraches

Thailand

Ha Nguyen

Australia

Christian Hans Nickel

Switzerland

Wendy Nicklin

Canada

Albert Nienhaus

Germany

Julius Ngigi Njogu

Kenya

Barnabas Njozing

Kenya

Lungiswa Nkonki

South Africa

Anne Nolan

Ireland

Carl Erik Nord

Sweden

Shane Norris

South Africa

Matthias Nuebling

Germany

Syed Nurulain

United Arab Emirates

Mads Nybo

Denmark

Jennifer Nyoni

Congo

Maeve O'Beirne

Canada

Melissa O'Connor

United States of America

Paul O'Connor

Ireland

Solomon Odafe

Nigeria

Doris Oestergaard

Denmark

Uchenna Ofoma

United States of America

Tinuade Ogunlesi

Nigeria

Cath O'Halloran

United Kingdom

Blythe O'Hara

Australia

Pamela Ohman Strickland

United States of America

Kentaro Okazaki

Japan

Edward Okeke

United States of America

Jiro Okochi

Japan

Roland Okoro

Nigeria

Ijeoma Okoronkwo

Nigeria

Oladepo Oladimeji

Nigeria

Kevin O'Leary

United States of America

Martine Oleche

Kenya

Oystein Evjen Olsen

Norway

Kari Olson

United States of America

Lars-Eric Olsson

Sweden

Bolajoko O Olusanya

Nigeria

Joyce O'Mahony

Canada

Joseph Onakewhor

Nigeria

Ciaran O'Neill

Ireland

Chima Onoka

Nigeria

Maricianah Atieno Onono

Kenya

Eberechukwu Onukwugha

United States of America

Wija Oortwijn

Netherlands

Carole Orchard

Canada

Dermot O'Reilly

United Kingdom

Joanna Orne-Gliemann

France

Emanuel Orozco

Mexico

Miguel Ortega

Spain

Herwig Ostermann

Austria

Margareta Östman

Sweden

Marielle Ouwens

Netherlands

Elizabeth Owiti

Kenya

Jane Padmore

United Kingdom

Raj Padwal

Canada

Kathleen Page

United States of America

Neil Pakenham-Walsh

United Kingdom

Simon Palfreyman

United Kingdom

Saseendran Pallikadavath

United Kingdom

Alexis Palmer

Canada

Pedro L Pancorbo-Hidalgo

Spain

Arvind Pandey

India

George Papachristou

Greece

Evelina Pappa

Greece

Bonny Parkinson

Australia

Sonak Pastakia

Kenya

Brijesh Patel

United Kingdom

James Paturas

United States of America

Milena Pavlova

Netherlands

Teresa Pawlikowska

Ireland

Sallie Pearson

Australia

David Peiris

Australia

Jean-Francois Pelletier

Canada

James Peltier

United States of America

Mikko Peltola

Finland

Jose Pereira

Canada

Julian Perelman

Portugal

Rafael Perera

United Kingdom

Marco Peres

Australia

Shoa-Jen Perng

Taiwan

Brian Perron

United States of America

Stephen Persell

United States of America

David Peters

United States of America

Stefan Peterson

Sweden

Dennis Petrie

Australia

Konstantinos Petsios

Greece

Isabelle Peytremann-Bridevaux

Switzerland

Holger Pfaff

Germany

Khanh Toan Pham

Viet Nam

Mai Pham

Australia

Mit Philips

Belgium

Alexandra Philipsen

Germany

Christine Phillips

Australia

James Phillips

United States of America

Rebecca Phillips

Australia

Anna Pierce

Australia

Ilkka Pietilä

Finland

Claudia Pileggi

Italy

Richard Pinder

United Kingdom

Hilary Pinnock

United Kingdom

Rogerio Pinto

United States of America

Anne Marie Plass

Netherlands

Diane Playford

United Kingdom

Thomas Plochg

Netherlands

Michael Polen

United States of America

Alex Pollock

United Kingdom

Kaja Polluste

Estonia

Marie-Pascale Pomey

Canada

Aron Frederik Popov

Germany

Cynthia Porter

Australia

Kevin Pottie

Canada

Byron Powell

United States of America

Wasana Prasitsuebsai

Thailand

Susan Pressler

United States of America

Matt Price

United States of America

Janice Probst

United States of America

Denis Protti

Canada

Janet Prvu Bettger

United States of America

Enriqueta Pujol-Ribera

Spain

Sarah Purdy

United Kingdom

Satvinder Purewal

United Kingdom

John Puvimanasinghe

Switzerland

Harith Qazaz

Iraq

Xuezheng Qin

China

Hude Quan

Canada

Wilm Quentin

Germany

Denise Quigley

United States of America

Julie Quinlivan

Australia

Terry Quinn

United Kingdom

Kristine Qureshi

United States of America

Jean-Sebastien Rachoin

United States of America

Laurie Rack

United States of America

Kavita Radhakrishnan

United States of America

Tapan Rai

Australia

Ravi Ram

Kenya

Ambili Ramachandran

United States of America

Erin Raney

United States of America

Krishna Rao

India

Maria Raven

United States of America

Marcus Redley

United Kingdom

Sabi Redwood

United Kingdom

Jens Peter Reese

Germany

Sandra Regan

Canada

Enrique Regidor

Spain

Stewart Reid

Zambia

Elizabeth Reifsnider

United States of America

Siobhan Reilly

United Kingdom

William Reinke

United States of America

William Renwick

Australia

Andrea Reupert

Australia

Debra Revere

United States of America

Louis Reynolds

South Africa

Matthew Reynolds

United Kingdom

Wadih Rhondali

France

Raul Ribeiro

United States of America

Walter Ricciardi

Italy

Jennifer Ridgeway

United States of America

Arthorn Riewpaiboon

Thailand

Susan Rifkin

United Kingdom

Nicola Ring

United Kingdom

Torsten Risor

Norway

Laetitia Rispel

South Africa

Christine Roberts

Australia

Andrej Robida

Slovenia

William Robiner

United States of America

Louise Robinson

United Kingdom

Suzanne Robinson

United Kingdom

Jaime Robledo

Colombia

Michael Robling

United Kingdom

Silvana Robone

Italy

Paul Robyn

Germany

Peter Rockers

United States of America

Anne Elizabeth Rogers

United Kingdom

Martin Roland

United Kingdom

Giulia Romano

Italy

Ulrich Ronellenfitsch

Germany

Katrina Aminah Ronis

Pakistan

Rob Roseby

Australia

Anne Rositch

United States of America

Charles Rosser

United States of America

Walter Rosser

Canada

Andrea Rotolo

Italy

Michael Rotondi

Canada

Libby Roughead

Australia

Greg Rowles

Australia

Kevin Rowley

Australia

Anthony Rudd

United Kingdom

Michael Rupp

United States of America

George Rutherford

United States of America

Eugene Ruzagira

Uganda

Bridget L. Ryan

Canada

Wael Sabbah

United Kingdom

Kate Sabot

United Kingdom

Cornelia Sack

Germany

Mitsuhiro Sado

Japan

Salim Sadruddin

Canada

Sami Saeed

Pakistan

Anju Sahay

United States of America

Olivier Saint Jean

France

Kenneth Sakauye

United States of America

Sanna Salanterä

Finland

Luis Salvador-Carulla

Spain

Domenico Salvatore

Italy

Nikolaos Samaras

Switzerland

Neelofar Sami

Pakistan

Emilia Sanchez

Spain

Magnus Sandberg

Sweden

Ryan Sandefer

United States of America

Felipe Sandoval

Japan

Hogne Sandvik

Norway

Gabriel Sanfélix-Gimeno

Spain

Claudia Sanmartin

Canada

Urmimala Sarkar

United States of America

Malabika Sarker

Bangladesh

Faruk Sarkinfada

Nigeria

Evelyn Sarnes

United States of America

Azusa Sato

United Kingdom

Yoshie Satoru

Japan

Mitzi Saunders

United States of America

Gianluigi Savarese

Italy

Dennis Scanlon

United States of America

Hendrik Simon Schaaf

South Africa

Luciene Cardoso Scherer

Brazil

Stephanie Schim

United States of America

Michaela Louise Schiøtz

Denmark

Niklas Schmedt

Germany

Eric Schneider

United States of America

Pia Schneider

United States of America

Ralph Schneider

Germany

Isabelle Scholl

Germany

Jos Schols

Netherlands

Jonas Schreyögg

Germany

Frederik Schut

Netherlands

Matthias Schützwohl

Germany

Rene Schwendimann

Switzerland

David Sclar

United States of America

Holly Seale

Australia

Federica Secci

United Kingdom

Chiara Seghieri

United Kingdom

Hildegard Seidl

Germany

Hanna Seidling

Germany

Andreas Seim

Norway

Innocent Semali

Tanzania

Susan Semple

Australia

Carlo Senore

Italy

Timo Seppälä

Finland

Ian Seppelt

Australia

Walter Sermeus

Belgium

Michal Shani

Israel

Charles Shaw

United Kingdom

Rod Sheaff

United Kingdom

Glenn Shedd

United States of America

Yuyan Shi

United States of America

Supriya Shore

United States of America

Elisa Sicuri

Spain

Peter Sidebotham

United Kingdom

Kristi Sidney

Sweden

Inger M. D. Siemsen

Denmark

Marc Silverstein

United States of America

Eva Silvestre

United States of America

Daudi Simba

Tanzania

Douglas Simkiss

United Kingdom

Steffen T. Simon

Germany

Tamara Simon

United States of America

Joanie Sims-Gould

Canada

Edina Sinanovic

South Africa

Timo Sinervo

Finland

Elvira Singh

South Africa

Harsimran Singh

United States of America

Jeshika Singh

United Kingdom

Carol Sinnott

Ireland

Heather Sipsma

United States of America

Olga Siskou

Greece

Susan Slaughter

Canada

Derek Smith

Australia

Garry Smith

Canada

Neale Smith

Canada

Scott Smith

United States of America

Tomotaka Sobue

Japan

Dominique Somme

France

David Sommerfeld

United States of America

Werner Soors

Belgium

Jacob Thorsted Sorensen

Denmark

Joann Sorra

United States of America

Paulo Sousa

Portugal

Danielle Southern

Canada

Adedoyin Soyibo

Nigeria

Mark Speechley

Canada

Stuart Speedie

United States of America

Anne Spencer

United Kingdom

Pamela Spencer

United States of America

Joanne Spetz

United States of America

Jean Spinks

Australia

Matthew Spittal

Australia

Catherine Spooner

Australia

Peter Spronk

Netherlands

Alessandro Squizzato

Italy

Gaby Sroczynski

Austria

Sarah Ssali

Uganda

Brendan Stack

United States of America

Anthony Staines

Ireland

John Stanback

United States of America

Tom Stargardt

Germany

J Stausberg

Germany

Tore W Steen

Botswana

Katharina Viktoria Stein

Austria

Laura Steinhardt

United States of America

Fiona Stevenson

United Kingdom

Alistair Stewart

New Zealand

Derek Stewart

United Kingdom

Joanna Stewart

New Zealand

Andrew Stoddart

United Kingdom

Emma Stokes

Ireland

Christian Stone

United States of America

Nick Stone

Australia

Roslyn Stone

United States of America

Marianne Storm

Norway

Judith Strawbridge

Ireland

Karl Stroetmann

Denmark

Johanita Strumpher

South Africa

Matthew Studenski

United States of America

Reijo Sund

Finland

Wayne Sunman

United Kingdom

Rosa Sunol

Spain

Tarja Suominen

Finland

Esther Suter

Canada

Rinku Sutradhar

Canada

Igor Svab

Slovenia

Sjoerd Sytema

Netherlands

David Szwajcer

Canada

Yayoi Takezako

Japan

Michael Tam

Australia

Giorgio Tamburlini

Italy

Miho Tanaka

United States of America

Ryan Tandjung

Switzerland

Yuexin Tang

United States of America

Balamurugan Tangiisuran

Malaysia

Sripen Tantivess

Thailand

Sihai Tao

China

Ruth Tappen

United States of America

Carolyn Tarrant

United Kingdom

Siegrid Tautz

Germany

Paula Tavrow

United States of America

Lee Taylor

Australia

Felicia Taylor Waller

United States of America

Gary Teare

Canada

Conor Teljeur

Ireland

Marleen Temmerman

Belgium

Cheong Lieng Teng

Malaysia

Flora Fang-Hwa Teng

Canada

Jeremy Theal

Canada

Horst Thiermann

Germany

Amardeep Thind

Canada

Sandra Thompson

Australia

Richard Thomson

United Kingdom

William Murray Thomson

New Zealand

Kevan Thorley

United Kingdom

Colin Thunhurst

United Kingdom

Gustav Tinghög

Sweden

Giuliano Tocci

Italy

Stephanie Topp

Zambia

Kwasi Torpey

Nigeria

Nassera Touati

Canada

Sarah Tougher

Uganda

Duong Tran

Australia

Gerd Tranø

Norway

Marta Trapero-Bertran

Spain

Joanne Travaglia

Australia

Ursula Trummer

Austria

Miao-Yu Tsai

Taiwan

Min-Fu Tsan

United States of America

Fu-Min Tseng

United Kingdom

Jennifer Tsui

United States of America

Hong Anh Tu

Netherlands

Prosper Tumusiime

Zimbabwe

Risto Tuominen

Finland

Janet Turan

United States of America

Erika Turkstra

Australia

Joanne Turnbull

United Kingdom

Rebecca Turner

United Kingdom

Simon Turner

United Kingdom

Patrick Twomey

United Kingdom

Liina-Kaisa Tynkkynen

Finland

Shahadat Uddin

Australia

Tuesday Udell

Australia

Maduka Ughasoro

Nigeria

Kingsley Nnanna Ukwaja

Nigeria

Noortje Uphoff

United Kingdom

Christine Urquhart

United Kingdom

V Anantharaman

Singapore

Per Valle

Norway

Bart Van Den Bemt

Netherlands

Geert Van Der Sluis

Netherlands

Sabine Van Der Veer

Netherlands

Karen Van Der Veken

Belgium

Philip Van Der Wees

Netherlands

Ineke Van Der Wulp

Netherlands

Malou Van Greuningen

Netherlands

Mark Van Ommeren

Switzerland

Anneloes Van Staa

Netherlands

Cornelis Johannes Van Stralen

Brazil

Zohreh Vanaki

Iran

Kris Vanhaecht

Belgium

Felipe Vasquez Lavin

Chile

Özalp Vayvay

Turkey

William Vega

United States of America

Marit B. Veierød

Norway

Paula Veiga

Portugal

Else Vengnes Grue

Norway

Edison Vidal

Brazil

Edgar Vieira

United States of America

Reinhold Vieth

Canada

Rebecca Vigen

United States of America

Irene Vikman

Sweden

Tereza Villa

Brazil

Jesus Villar

Spain

Varsha Vimalananda

United States of America

Pekka Virtanen

Finland

Gunn Elisabeth Vist

Norway

Peter Vogt

Germany

Kranti Vora

India

Francis Wafula

Kenya

Glenn Wagner

United States of America

Olive Wahoush

Canada

Christine Walker

Australia

Yuri Walker

United States of America

Allan Walkey

United States of America

John Walley

United Kingdom

Scott Walter

Australia

Merrilyn Walton

Australia

Lu Wang

Canada

Wei Wang

Australia

Patrick Waterson

United Kingdom

Verity Watson

United Kingdom

Susan Watts

United Kingdom

Robert Wears

United States of America

Cynthia Webster

Australia

Kristina Weeks

United States of America

William Weeks

United States of America

Matthias Weigl

Germany

Claus Wendt

Germany

Rhay-Hung Weng

Taiwan

Erik L Werner

Norway

Margareta Westerbotn

Sweden

Björn Wettermark

Sweden

Jacquie White

United Kingdom

Dean Whitehead

New Zealand

Susan Whiting

Canada

Andreas Isidorus Wierdsma

Netherlands

Helena Wigert

Sweden

Siri Wiig

Norway

Janice Wiley

Australia

Torsten Wilhelm

Germany

Scott Wilkes

United Kingdom

Joyce Wilkinson

United Kingdom

Helen Williams

United Kingdom

Judith Williams

United Kingdom

Sarah Willis

United Kingdom

Andrew Wilson

United States of America

Michael Wilson

Canada

Anders Wimo

Sweden

Haije Wind

Netherlands

Yaroslav Winter

Germany

Torben Wisborg

Norway

Meg Wise

United States of America

Adrienne Withall

Australia

Mirkuzie Woldie

Ethiopia

Ruth Wolever

United States of America

Charles Wolfe

United Kingdom

Miranda Wolpert

United Kingdom

Albert Wong

Netherlands

Eliza Wong

Hong Kong

Geoff Wong

United Kingdom

Li Ping Wong

Malaysia

Richard Woodman

Australia

Nancy Woods

United States of America

Philip Worrall

United Kingdom

Linda Worrall-Carter

Australia

Pamela Wright

Netherlands

Shih-Wang Wu

Taiwan

Tai-Hsi Wu

Taiwan

Gwenllian Wynne-Jones

United Kingdom

Kaspar Wyss

Switzerland

Fei Yan

China

Che-Ming Yang

Taiwan

Wei Yang

United Kingdom

Bobbi Jo Yarborough

United States of America

Maurice Yé

Burkina Faso

Veerasamy Yengopal

South Africa

Doris Young

Australia

Jane Young

Australia

Tracey Young

United Kingdom

Yunxian Yu

China

Elaine Yuen

United States of America

Claudio Zaugg

Switzerland

Dimitrios Zavras

Greece

Teresa Zayas- Cabán

United States of America

John Zeber

United States of America

Peter Zed

Canada

Lingxia Zeng

China

Mikio Zeniya

Japan

Eyob Zere

Zimbabwe

Siyan Zhan

China

Wei-Hong Zhang

Belgium

Xinping Zhang

China

Yuejen Zhao

Australia

Zhongliang Zhou

China

Junya Zhu

United States of America

Walter Zidek

Germany

Renate Zilkens

Australia

David Zimmer

United States of America

Hans Zoellner

Australia

Pascal Zurn

Switzerland

Nicholas Zwar

Australia

Nicolien Zwijnenberg

Netherlands

## Pre-publication history

The pre-publication history for this paper can be accessed here:

http://www.biomedcentral.com/1472-6963/14/67/prepub

